# Direct Growth of Feather-Like ZnO Structures by a Facile Solution Technique for Photo-Detecting Application

**DOI:** 10.1186/s11671-017-2252-0

**Published:** 2017-08-10

**Authors:** Yurong Jiang, Xingbing Liu, Fangmin Cai, Hairui Liu

**Affiliations:** 1 Henan Key Laboratory of Photovoltaic Materials, College of Physics and Materials Science, Henan Normal University, Xinxiang, 453007 China; 2 School of Computer and Information Engineering, Henan Normal University, Xinxiang, China

**Keywords:** Photo-response, Nanostructures, Feather-like hierarchical structures, Successive ionic layer adsorption and reaction

## Abstract

The feather-like hierarchical zinc oxide (ZnO) was synthesized via successive ionic layer adsorption and reaction without any seed layer or metal catalyst. A possible growth mechanism is proposed to explain the forming process of ZnO feather-like structures. Meanwhile, the photo-electronic performances of the feather-like ZnO have been investigated with the UV-vis-NIR spectroscopy, I-V and I-tmeasurements. The results indicate that feather-like ZnO hierarchical structures have good anti-reflection and excellent photo-sensitivity. All results suggest that the direct growth processing of novel feather-like ZnO is envisaged to have promising application in the field of photo-detector devices.

## Background

Zinc oxide (ZnO) is a very versatile material due to its wide bandgap (~3.37 eV) and large exciton binding energy, up to 60 meV, which allow the fabrication of UV [1, 2] and blue light-emitting diode [3]. In recent years, intensive efforts have been put in the exploration of photodetectors [4, 5] based on the three-dimensional (3D) ZnO architectures with the micrometer- and nanometer-scale building blocks. Compared with mono-morphological ZnO structures, 3D hierarchical ZnO structures possess a large surface area which could facilitate the adsorption of light. Generally, 3D hierarchical ZnO structures such as flower-like structures [6], texture [7], nanotubes [8], and dendritic-like [9] and feather-like [10] structures exhibit outstanding optical [11], electronic [12], catalytic properties [9] and thus have many potential applications in solar cells, gas sensors, photo-catalysts, and other fields. To synthesize hierarchical ZnO structures, various physical, chemical [13], and electrochemical [14] methods have been employed. Among them, the hydrothermal/solvothermal method [15] is very popular because of its handy and large area preparation. However, these methods often require a seed layer and metal catalysts. ZnO seed layer growth may already have a well control for the ZnO nanostructure growth, which normally needs to be annealed with a high temperature or complicated vacuum equipments [16]. In addition, using a seed layer and metal catalysts could make the synthesis procedure more complex and introduce impurities which influence the properties of the ZnO structure.

Therefore, it still remains an enormous challenge to develop a facile room-temperature method that needs not any seed layer or metal catalyst for producing hierarchical ZnO structures.

Herein, in this work, a new attempt was made to prepare ZnO hierarchical structures, which was used without any seed layer or metal catalyst based on successive ionic layer adsorption and reaction (SILAR) processing. The novel and unusual feather-like ZnO hierarchical structures were obtained for the first time based on SILAR at room temperature. A possible mechanism was proposed to explain the growth process of the ZnO feather-like structures. In addition, the photoelectric properties of the feather-like ZnO/p-Si heterojunctions had been investigated, and the results indicate that feather-like ZnO nanostructures have excellent anti-reflection characteristics and good photosensitivity, which suggests that these hierarchical structures have a potential application in the photo-electronic devices.

## Methods

First Si (100) substrates were ultrasonically cleaned for 10 min in ethanol. Second, 0.01 mol of zinc acetate (Zn(CH_3_COO)_2_) was dissolved into 100 mL of deionized water, then ammonia hydroxide was added into the solution until its pH was around 11, to form a uniform transparent solution under stirring, which is the precursor solution of feather-like ZnO. Afterward, silicon wafer was dipped into the predecessor solution for 30 s, and the ion complex was absorbed into the Si substrate, then the Si substrate was taken out and put into deionized water for 20 s and was washed with ultrapure water for 20 times to remove impurities such as unconsolidated zinc hydroxide (Zn(OH)_2_). Finally, the samples were put into deionized water with 90 °C for 1 min; in this step, the unreacted ion complex and zinc hydroxide which had been absorbed can be resolved into pure ZnO. In a typical SILAR experiment, we circulated the above steps for 20 times. The crystal structures of feather-like ZnO were characterized by X-ray diffraction (XRD) and energy disperse spectrometer (EDS). The surface morphology was investigated by scanning electron microscopy (SEM) and transporting electron microscopy (TEM). Furthermore, we also analyzed *I*-*V* and *I*-*t* characteristics of feather-like ZnO/p-Si. In order to measure the photo-diodes characteristics, the electrode of 12-nm semitransparent Cu film was deposited on the ZnO/p-Si by the thermal evaporation masked with an area of 5 mm × 5 mm. The schematic of diode is shown in Fig. 4c.

## Results and discussion

**Fig. 1:**
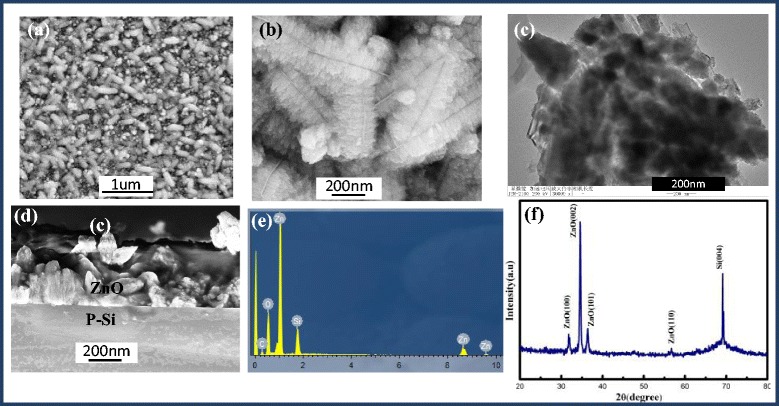
**a**, **b** The SEM images of feather-like ZnO grown on silicon. **c** The TEM image of individual feather-like ZnO. **d** The cross-sectional SEM image of feather-like ZnO/p-Si. **e** The EDS analysis of the ZnO/p-Si, indicating that the predominant composition is Zn. **f** XRD patterns of feather-like ZnO/p-Si

**Fig. 2:**
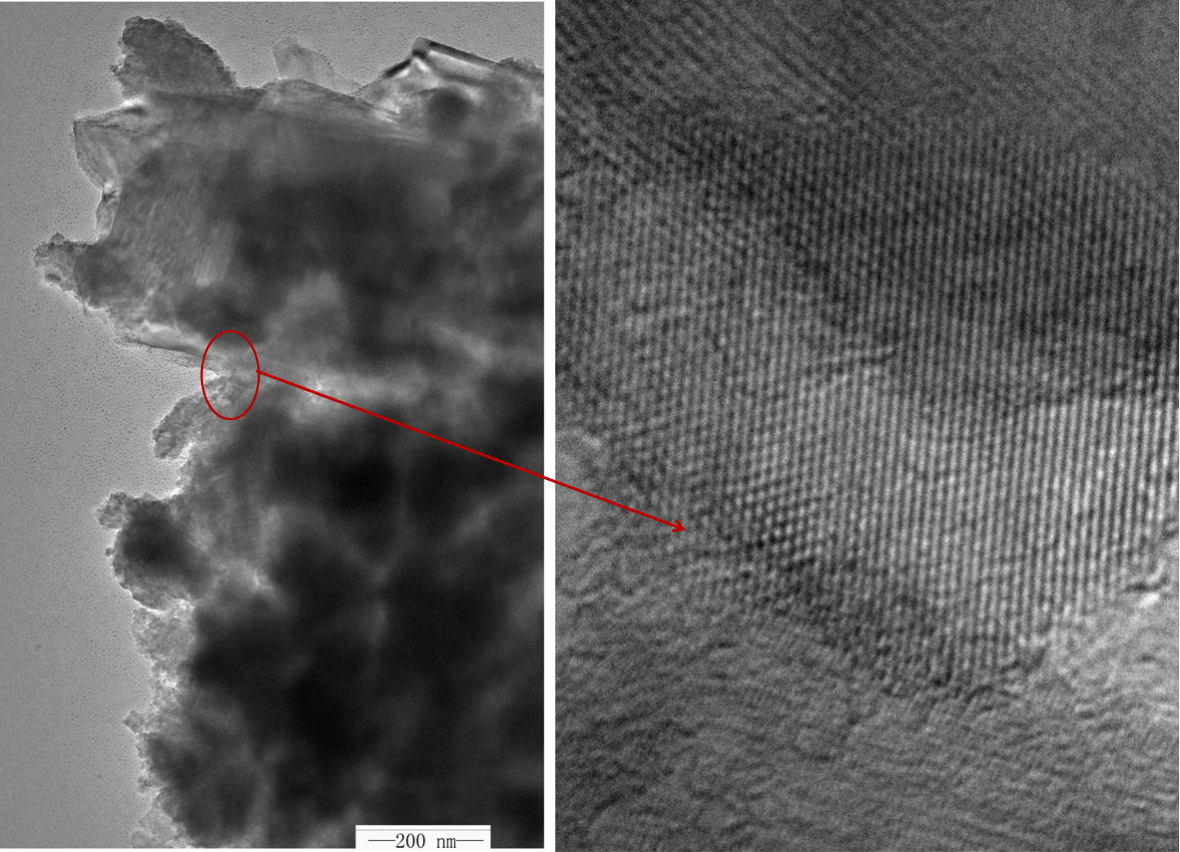
TEM images of a hierarchical ZnO structure segment

Figure 1a shows that ZnO has feather-like morphology, which is novel and unusual. The longitudinal length of feather-like structures varies between 300 and 800 nm, and its lateral length is different from 200 to 400 nm. The magnified SEM image in Fig. 1b shows that the hierarchical structures are obtained. Meanwhile, the branches of feather-like 3D structures are interestingly assembled perpendicularly to the nano-sheet trunks. Figure 1c shows the TEM image of an individual hierarchical structure. The dark dots and translucent plate correspond to the branches and the nano-sheet trunk. Because the size of feather-like ZnO is beyond 200 nm, the lattice fringe could not be revealed. Figure 2 show the typical TEM images of a nanorod segment form the ZnO feathers, it proves the nanorod is a single crystal.

Figure 1e shows the peaks of EDS in which only Zn, O, C, and Si were found in our sample, which indicates that the process of SILAR is successful to deposit pure ZnO onto silicon. The XRD (Fig. 1e) reveals the crystal structure and phase purity of the ZnO hierarchical structures. All the diffraction peaks of the products match very well with those of wurtzite ZnO (JCPDS file 36-1451), as well as a dominant diffraction peak corresponding to the p-Si (400). No diffraction peaks from other impurities are found in the spectrum; the result indicates that the structure is pure hexagonal wurtzite ZnO. Moreover, the intensity of peak (002) is rather higher than peaks (100) and (101); this shows that the crystalline is along the (002) axis preferred orientation. The sharp diffraction peaks reveal that ZnO have high crystal structure of pure quality.

**Fig. 3:**
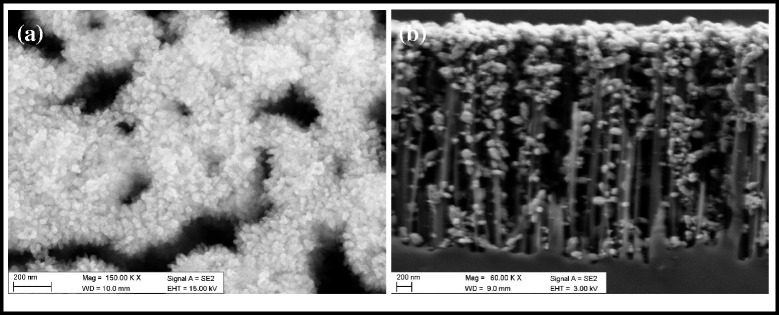
SEM images of ZnO grown on silicon nanowires: **a** morphology and **b** cross section

It should be mentioned here that no ZnO hierarchical structures are found even though the reaction is carried out under the same environment when using Si nanowires with all crystal directions replacing Si (100) substrates (shown as Fig. 3). The results indicate that the crystal direction plays a key role in the nucleation and growth of ZnO hierarchical structure.

**Fig. 4:**
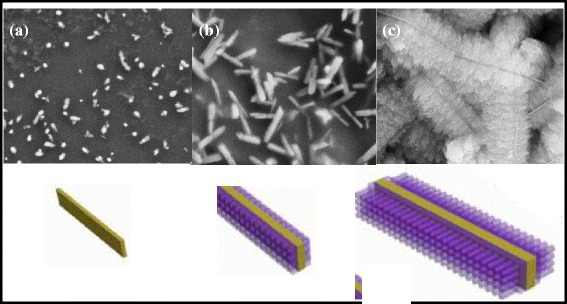
The schematic diagram of formation processes for ZnO hierarchical structures: **a** the trunk formation of ZnO nano-sheets; **b** secondary heterogeneous nucleation and growth of branches; **c** the continuously growth of primary nano-sheets and secondary nano-branches constructs the feather-like ZnO hierarchical structures

On the basis of the above results, it can be speculated that the feather-like ZnO hierarchical structures were synthesized via a two-stage nucleation-growth process. Figure 4 shows the schematic diagram describing the formation processes of ZnO hierarchical structures. First, ammonia hydroxide is used to provide hybroxyl anions (OH^−^) which increases the pH of reaction solution and the alkalinity of the reaction solution, then the Zn(OH)_4_^2−^ ions are obtained. Upon the dehydration of Zn(OH)_4_^2−^ ions, Zn(OH)_4_^2−^ ions are adsorbed onto the Si substrate and subsequently dissolved to form homogeneous ZnO nuclei followed by the water bath at 90 °C [17]. During this process, the trunk formation of ZnO nano-sheets with {110} planar surface at the initial stage can be ascribed to the excess OH^−^ ions and abundant Zn(OH)_4_^2−^ ions (shown as Fig. 4a), which can stabilize the surface charge and the structure of Zn (001) surface to some extent, allowing fast growth along the [100] direction [18]. Second, the surface of the primary ZnO nano-sheets trunk formed during the initial growth stage has many crystalline boundaries which contain more defects than other regions. These defects on the surface of trunk provide active sites for secondary heterogeneous nucleation and growth of branches (shown as Fig. 4b). Finally, the continuously growth of primary nano-sheets and secondary nano-branches constructs the feather-like ZnO hierarchical structures (shown as Fig. 4c).

**Fig. 5:**
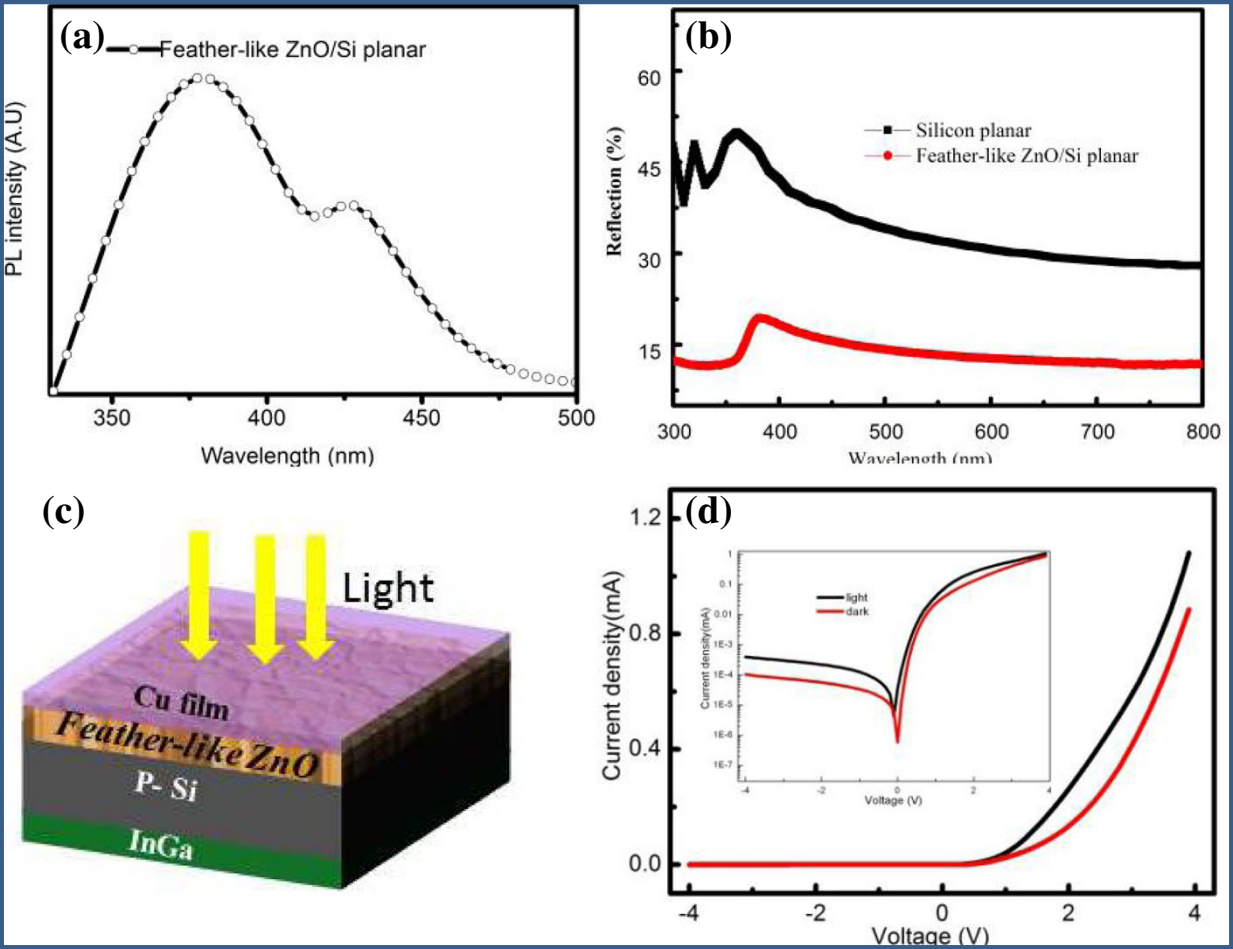
**a** PL spectrum of feather-like ZnO. **b** Reflection spectra of ZnO/Si and Si planar. **c** The schematic of feather-like ZnO/Si photo-diodes. **d** *I*-*V* curves of feather-like ZnO/Si; the inset of **d** is the lnI-*V* curves

To investigate the optical properties of feather-like ZnO, the room-temperature PL was obtained by using a He–Cd laser (*λ* = 325 nm) as the excitation source as shown in Fig. 5a. Two emission peaks are apparently observed. The first emission band at 384 nm is obviously caused by the excitations, which can be attributed to the UV near-band edge emission [18]. Meanwhile, it is visualized that the weaker visible emission appeared by a broad emission band at 443 nm in the green region, revealing their collective optical properties. The irradiative recombination of a photo-generated non-equilibrium carriers occupying the oxygen vacancy may give rise to the green peak would be the existence of oxygen vacancies in the films [19].

Figure 5b shows reflection of the feather-like ZnO/Si and planar Si measured by UV-vis-NIR spectroscopy. It shows that reflection of feather-like ZnO/Si is obviously reduced compared with p-Si planar (from 40 to 10%), and a relatively low reflection in the range of 300 to 400 nm resulting from band-to-band absorption. The superior anti-reflection characteristics with an average reflection of less than 10% are observed for ZnO/Si in wavelengths shorter than 400 nm which is the optical bandgap of ZnO materials [20]. This result indicates that feather-like ZnO structures act as an excellent anti-reflection. Therefore, it has a potential application as the anti-reflection in solar cell.

Figure 5d shows the *I*-*V* curve of feather-like ZnO/p-Si heterojunction, which is measured in dark and under AM 1.5 sunlight respectively at room temperature. It shows rectifying behavior for the junctions indicating formation of a diode between ZnO and Si. The rectification ratio is as high as 535 at −1 V (1695 at −2 V) in a dark condition. This indicates that the rectifying behavior of ZnO/Si is quite excellent. Theoretically, the *I*-*V* relation for a heterojunction could be described as

1 $$ I={I}_0\left\{\exp \left[\frac{q\left(v-{IR}_{\mathrm{s}}\right)}{nKT}\right]-1\right\} $$

where *K* is the Boltzmann’s constant, *T* is the absolute temperature in Kelvin, *q* is the unit charge of a single electron, and *n* is the ideality factor. *R*_s_ is the series resistance of the diode, and *I*_0_ is the reverse bias saturation current represented. The behavior of the *I*-*V* curve can be partly explained by a band diagram based on the Anderson model [21]. Moreover, the ratio of photo current to dark current is ~90.24 under the reverse bias at −2 V bias, which suggests that this structure has an obvious photo-response behavior.

**Fig. 6:**
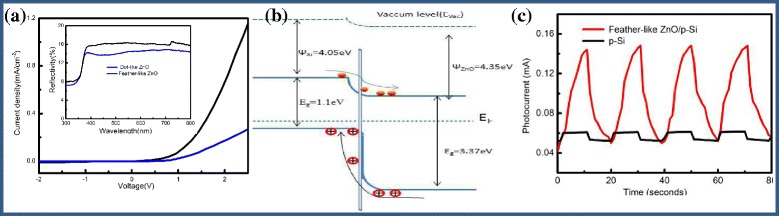
**a** *I*-*V* curves of feather-like ZnO/Si and nano-dot ZnO/p-Si; the insert is the reflection spectra. **b** The energy band diagram of ZnO/p-Si heterojunction. **c** *I*-*t* curves of feather-like ZnO/p-Si and p-Si planar structures

To confirm further that the present feather-like hierarchical structures offer the beneficial effect on rectifying characteristics, we have also measured the *I*-*V* characteristics of nano-dot-like ZnO/Si (Fig. 6a). The results indicate that feather-like hierarchical ZnO/Si had a better rectifying effect than nano-dot-like ZnO/Si. Therefore, the feather-like hierarchical ZnO could effectively suppress the charges recombination activity and enhance the rectifying effect.

The energy band diagram of ZnO/p-Si heterojunction was constructed at equilibrium shown as Fig. 6b. In this diagram, the electron affinities for ZnO and Si are taken as 4.35 and 4.05 eV, respectively.

The conduction band offset is ∆*E*_c_ = 0.3 eV, while the valence band offset is ∆*E*_v_ = 2.54 eV; thus, the conduction of holes dominates the forward *I*-*V* characteristic of the junction. The valence band offset is very large, there is a diffusion of electrons from n-ZnO to p-Si and diffusion of holes from p-Si to n-ZnO because electrons are minority carriers and holes are majority carriers in p-Si and electron are majority carriers and holes are minority carriers in n-ZnO. At low forward voltage, the current increases exponentially. Therefore, the forward *I*-*V* characteristics in Fig. 4d can be explained.

Figure 6c is the *I*-*t* curve of the feather-like ZnO/p-Si and p-Si planar structure when irradiated with 365-nm UV light at 1-V bias voltage. The response current (*I*_light_ = *I*_UV_ − *I*_dark_) in the ZnO/p-Si device is 0.10 mA, which is 90% enhancement as compared to Si planar device having a response current of 0.01 mA. The enhancement in the response current of ZnO/p-Si as compared to p-Si planar mainly could be due to the presence of ZnO/p-Si heterojuncton, which could fastly separate the generated carriers and reduce the recombination rate of photogenerated free charge carriers. The feather-like ZnO/p-Si device show a single exponential rise under illumination which can be attributed to the recombination of the electron-hole pairs. In Table 1, we reviewed all parameters from the two devices. As compared with bare Si planar, the sensitivity of the feather-like ZnO/Si structure has been improved nearly 10 times. Furthermore, as shown in Fig. 5c, their rise and decay times have been greatly increased for the feather-like ZnO/Si device which can be attributed to the recombination of holes-electrons. The results suggest that the feather-like hierarchical ZnO structures exhibit excellent sensitivity to UV light. These cyclic behaviors also reveal that both devices show highly repeatable photo-response with UV illumination.

**Table 1:** The photo-response parameters of the feather-like ZnO/p-Si and p-Si planar structures

Samples	*I*_dark_ (mA)	*I*_light_ (mA)	Sensitivity	*τ*_*g*_ (s)	*τ*_*d*_ (s)
Feather-like ZnO/p-Si	0.048	0.159	2.3	11	9
p-Si	0.051	0.0612	0.22	2.2	2.1

## Conclusions

Feather-like hierarchical ZnO structures were successfully synthesized without any seed layer or metal catalyst by a facile SILAR technique at room temperature. The probable mechanism of a two-stage nucleation-growth process had been proposed. Meanwhile, the feather-like ZnO possesses excellent anti-reflection, good photo-response, and enhanced UV photocurrent. All enhanced characteristics are attributed to the presence of novel feather-like ZnO; this hierarchical ZnO structures probably have potential application in photo-detector devices.
